# Frequent longitudinal blood microsampling and proteome monitoring identify disease markers and enable timely intervention in a mouse model of type 1 diabetes

**DOI:** 10.1007/s00125-025-06502-7

**Published:** 2025-08-04

**Authors:** Anirudra Parajuli, Annika Bendes, Fabian Byvald, Virginia M. Stone, Emma E. Ringqvist, Marta Butrym, Emmanouil Angelis, Sophie Kipper, Stefan Bauer, Niclas Roxhed, Jochen M. Schwenk, Malin Flodström-Tullberg

**Affiliations:** 1 Department of Medicine Huddinge, Karolinska Institutet, Stockholm, Sweden; 2 Science for Life Laboratory, Department of Protein Science, KTH Royal Institute of Technology, Solna, Sweden; 3 TUM School of Computation, Information and Technology, Technical University of Munich, Munich, Germany; 4 Helmholtz AI, Munich, Germany; 5 Department of Intelligent Systems, KTH Royal Institute of Technology, Stockholm, Sweden; 6 MedTechLabs, Karolinska University Hospital, Solna, Sweden

**Keywords:** Biomarkers, Coxsackievirus B, Disease intervention, Disease prediction, Disease trigger, Dried blood spots, Enterovirus, Immune-mediated diseases, Machine learning, Microsampling, Proteomics, Proximity extension assay, Screening, Type 1 diabetes

## Abstract

**Aims/hypothesis:**

Type 1 diabetes manifests after irreversible beta cell damage, highlighting the crucial need for markers of the presymptomatic phase to enable early and effective interventions. Current efforts to identify molecular markers of disease-triggering events lack resolution and convenience. Analysing frequently self-collected dried blood spots (DBS) could enable the detection of early disease-predictive markers and facilitate tailored interventions. Here, we present a novel strategy for monitoring transient molecular changes induced by environmental triggers that enable timely disease interception.

**Methods:**

Whole blood (10 μl) was sampled regularly (every 1–5 days) from adult NOD mice infected with Coxsackievirus B3 (CVB3) or treated with vehicle alone. Blood samples (5 μl) were dried on filter discs. DBS samples were analysed by proximity extension assay. Generalised additive models were used to assess linear and non-linear relationships between protein levels and the number of days post infection (p.i.). A multi-layer perceptron (MLP) classifier was developed to predict infection status. CVB3-infected SOCS-1-transgenic (tg) mice were treated with immune- or non-immune sera on days 2 and 3 p.i., followed by monitoring of diabetes development.

**Results:**

Frequent blood sampling and longitudinal measurement of the blood proteome revealed transient molecular changes in virus-infected animals that would have been missed with less frequent sampling. The MLP classifier predicted infection status after day 2 p.i. with over 90% accuracy. Treatment with immune sera on day 2 p.i. prevented diabetes development in all (100%) of CVB3-infected SOCS-1-tg NOD mice while five out of eight (62.5%) of the CVB3-infected controls treated with non-immune sera developed diabetes.

**Conclusions/interpretation:**

Our study demonstrates the utility of frequently collected DBS samples to monitor dynamic proteome changes induced by an environmental trigger during the presymptomatic phase of type 1 diabetes. This approach enables disease interception and can be translated into human initiatives, offering a new method for early detection and intervention in type 1 diabetes.

**Data and code availability:**

Additional data available at https://doi.org/10.17044/scilifelab.27368322. Additional visualisations are presented in the Shiny app interface https://mouse-dbs-profiling.serve.scilifelab.se/.

**Graphical Abstract:**

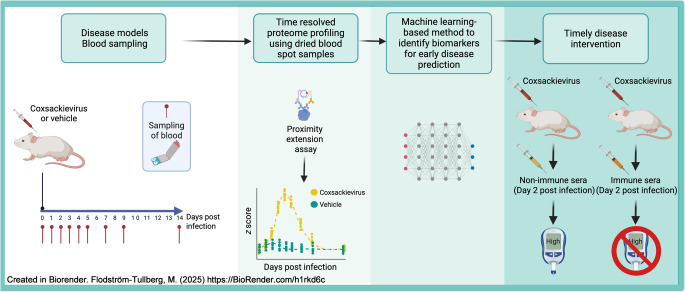

**Supplementary Information:**

The online version of this article (10.1007/s00125-025-06502-7) contains peer-reviewed but unedited supplementary material.


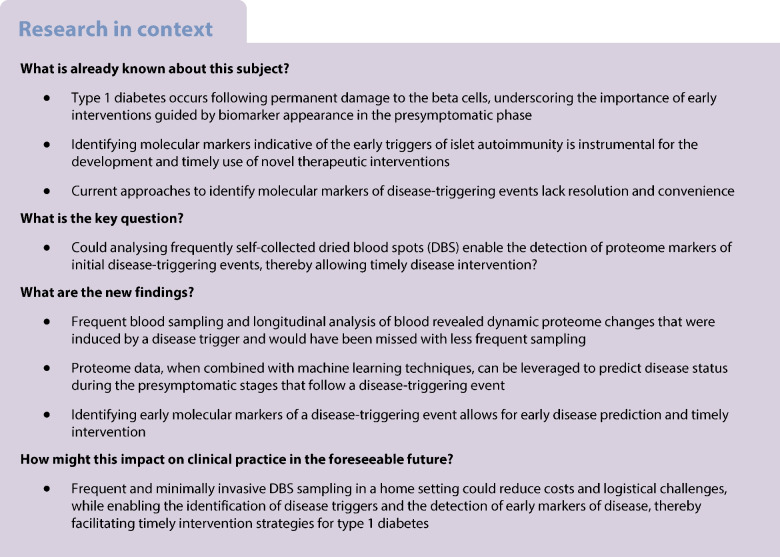


## Introduction

An essential goal of precision medicine is to prevent diseases before they manifest [1]. This relies on the identification of predictive biomarkers that can be detected in asymptomatic individuals. Existing molecular tools and approaches remain suboptimal for precise molecular monitoring of at-risk individuals. One key limitation is that current monitoring schemes cannot detect fluctuations in molecular markers that are transiently induced by a trigger of disease.

Type 1 diabetes is characterised by the loss of functional pancreatic beta cells and reduced insulin production. The autoimmune origin of type 1 diabetes is intimated by the appearance, approximately 6 months to a few years before disease onset, of islet reactive autoantibodies (AAbs). This occurs in over 95% of those who develop the condition. Additionally, autoreactive T cells are detected in the pancreas before clinical symptoms manifest [2]. Environmental factors play a role in mediating type 1 diabetes risk, but the exact triggers behind the break in immunological tolerance to the beta cells remain unknown [3]. Several studies suggest an enterovirus infection may precede the first appearance of islet AAbs [4]. Consequently, a well-timed antiviral therapy could intervene in the events that initiate islet autoimmunity and may also help preserve beta cell function at diagnosis [5]. Treatment with the T cell targeting anti-CD3 antibody (teplizumab) can delay disease onset for 2–3 years in islet AAb-positive individuals at very high-risk of type 1 diabetes [6–8]. Identifying those who have been infected by a potentially diabetogenic virus but not yet developed islet autoimmunity, or who are islet AAb positive and near disease onset, remains a significant and currently unresolved challenge.

A global analysis of proteins may offer valuable insights into different disease stages, disease progression and treatment responses [9]. Accordingly, advanced proteomic techniques may be leveraged for the detection of early stages of disease, ideally in easily accessible body fluids [10, 11]. Recent breakthroughs have enabled precise and high-throughput measurements of blood proteins [10, 12]. These cutting-edge techniques allow comprehensive proteome analysis and can identify subtle disease-related or predictive protein changes [13]. Despite new advances in detecting proteomic disease signatures [14], blood sampling often still necessitates visits to healthcare centres. This inconvenience frequently results in singular blood samples or lengthy timespans between samples (e.g. months to years) which miss rapid and transient proteome changes associated with the events that trigger disease and/or disease progression. Previous prospective studies (e.g. TEDDY and DIPP) identified serum proteome changes that are linked to the onset of islet AAbs or type 1 diabetes, but proteome profiles have not yet been correlated with a disease-triggering event [15, 16]. Overcoming the limitation of infrequent sampling is crucial for timely disease detection and the implementation of personalised disease interventions.

Promising remote sampling methods which could permit frequent self-sampling include the collection of dried blood spots (DBS). DBS samples can be shipped by regular mail to advanced laboratories. We recently demonstrated that at-home unsupervised volumetric DBS sampling by finger-pricking allows for the precise measurement of multiple circulating antibodies associated with COVID-19 [17]. Using state-of-the-art proteomic tools, we also showed that 10 µl of dried blood was sufficient to successfully quantify hundreds of circulating proteins [18]. Building on this, we hypothesised that quantitative analysis of frequently collected DBS can reveal circulating proteins or proteome signatures that are indicative of a disease-triggering event. In this study, we used experimental models of enterovirus-triggered type 1 diabetes [19–21] to investigate whether frequent blood microsampling and affinity-based proteomics capture changes in the circulating proteome that are induced by a disease trigger and whether this enables timely, molecularly informed interventions.

## Methods

### Animal husbandry and monitoring of animal health

NOD-Shi and *SOCS1* transgenic (SOCS-1-tg) NOD [19, 22] mice were bred in-house and maintained at the Karolinska University Hospital Huddinge, Stockholm, Sweden. Approval was received from the local ethics committee (Linköpings djurförsöksetiska nämnd, Dnr 9222-2019 and 04291-2024). Studies were conducted according to institutional guidelines and Swedish national laws. Mice were randomly allocated to treatment groups and researchers remained unblinded. Health status was closely monitored. See further details in ESM Methods.

### Virus infections and serum transfers

**Fig. 1:**
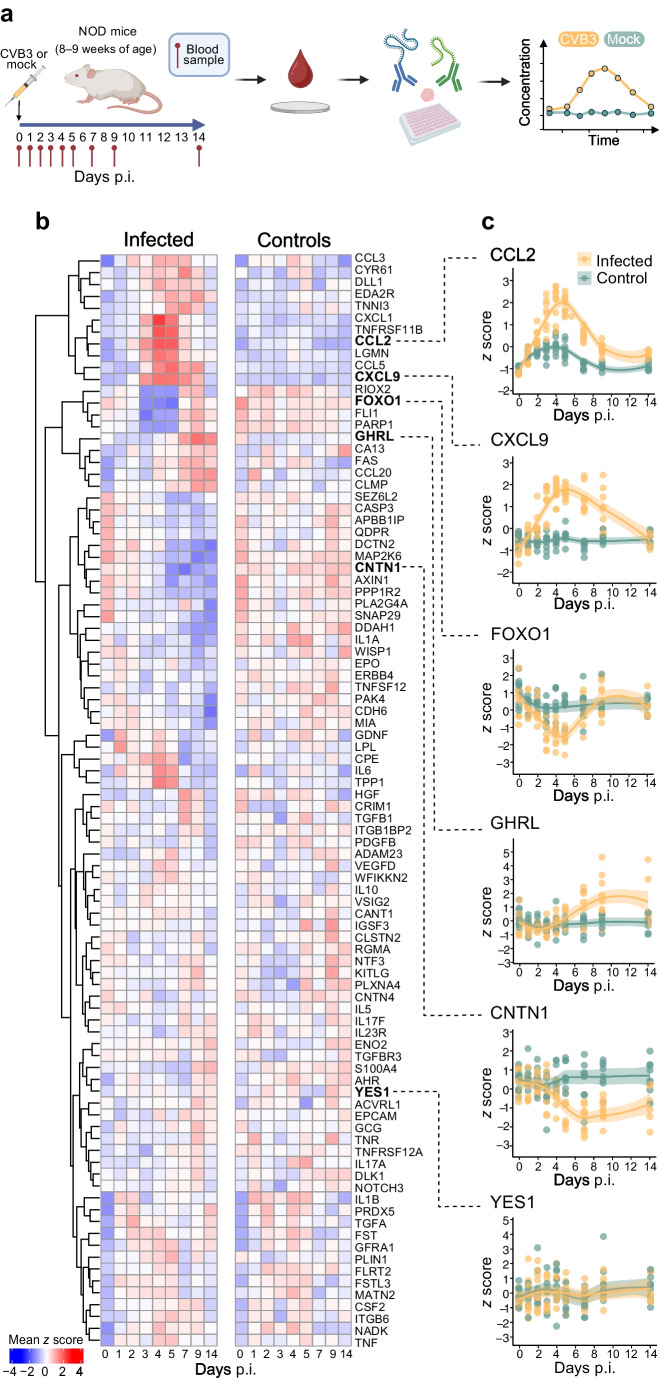
Longitudinal DBS protein profiling reveals dynamic proteome alterations in CVB3-infected NOD mice. (**a**) Schematic of study design. In two independent studies, NOD mice aged 8–9 weeks were infected with CVB3 (200 µl RPMI medium containing 10^5^ PFU CVB3, i.p.) or mock-infected (200 µl of RPMI medium). In the first study, the control and the CVB-infected groups consisted of four animals each and in the second study, the control group consisted of four animals and the CVB-infected group of five animals. A blood sample (5 µl) was collected from the tail vein at indicated time points and dispensed onto filter discs (Capitainer). Blood samples were eluted and proteins were measured using PEA (Olink), followed by data analysis. Created in BioRender. Byvald, F. (2024) https://BioRender.com/t83q649. (**b**) Heatmaps showing the mean protein levels (*z* score) per sampling day for each protein for the infected (left) and control (right) groups. The proteins are clustered based on the levels in the infected group. Red indicates a high *z* score and blue indicates a low *z* score. (**c**) Protein profiles for six proteins. Each dot represents a sample, and colour indicates if the included mouse belonged to the infected (yellow) or control (green) group. Smooth lines have been fitted to each group, with the 95% CI around the smooth line

Female NOD mice (8–9 weeks old) were infected with 10^5^ plaque forming units (PFU) of Coxsackievirus B3 (CVB3; Nancy) or vehicle (mock; RPMI media), by i.p. injection (total volume 200 µl). Blood samples were collected at the indicated time points until day 14 post infection (p.i.; Fig. 1a). Animals were classified as infected if they exhibited a reduction in blood glucose levels during the acute phase of infection (days 0–7 p.i.) and showed histological evidence of pancreatic damage at the end of the study. Based on these criteria, one animal from each infection experiment was excluded from the analyses (total *n *= 2). To produce immune sera, male and female NOD mice (11–12 weeks) were infected with CVB3 (5 × 10^4^ PFU; i.p. injection) and blood was collected 2 weeks p.i. by terminal heart puncture. Non-immune sera were collected from age-matched uninfected NOD mice. Male and female SOCS-1-tg mice (8–10 weeks old) were infected with CVB3 (10^5^ PFU; 200 µl/mouse; i.p. injection) and injected with pooled and heat-inactivated sera (inactivated at 56°C for 30 min; 200 µl/injection; i.p. route) on days 2 and 3 p.i.

### Blood collection, glucose measuring and monitoring of diabetes development

Blood was collected for proteome analysis from the tip of the tail. A sample of 5 µl was transferred onto paper-based sampling discs (Capitainer, 710-0020, Solna, Sweden) and left to dry until analysis (see ESM Methods). Blood glucose levels from tail vein blood were measured using a Bayer Contour XT blood glucose meter (Bayer, Basel, Switzerland). Diabetes was diagnosed when blood glucose levels were ≥18 mmol/l or after two consecutive daily measurements of 13–18 mmol/l.

### Histology and immunohistochemistry

Formalin-fixed, paraffin-embedded pancreas was sectioned (5 µm) and H&E stained (morphological assessments) or immunohistochemically stained for insulin and glucagon as previously described [19, 23] (see ESM Methods).

### Blood sample processing and proteomics assay

Briefly, DBS samples were eluted in PBS with 0.05% Tween 20 (Thermo Fisher Scientific, Stockholm, Sweden). The Olink Target 96 Mouse Exploratory panel was used to measure 92 circulating proteins from the eluates which were assessed by proximity extension assays (PEA). Linear and non-linear relationships between protein levels and days p.i. were assessed using generalised additive models (GAMs). See ESM Methods for detailed information.

### Statistical analysis

Statistical analyses were performed using Prism 10 software (version 10.3.1; GraphPad, La Jolla, CA) and R (version 4.4.1, https://www.R-project.org/) [24] unless otherwise stated. Plots were produced in Prism or R using the ggplot2 package (version 3.5.1, https://ggplot2.tidyverse.org) [25]. Data are expressed as mean ± SD. False discovery rate (FDR) correction of multiple testing was performed using the Benjamini-Hochberg/Bonferroni method. A nominal *p* value or FDR <0.05 was considered statistically significant. The reproducibility of DBS sampling and the proteomics assay process was tested in biological replicates collected concurrently (six samples from one mouse in Study 1; five samples from one mouse in Study 2), and in technical duplicates. See ESM Methods for details.

### Generation of a classifier to predict infection status

Machine learning (ML) techniques were used to generate a multi-layer perceptron (MLP) classifier to predict infection status. See ESM Methods for details.

### Web-based interface

The *shiny* package (version 1.8.1.1, https://shiny.posit.co/) [26] was used to create an interactive web interface. The app allows a protein-centric visualisation of the proteomics data with interactive heatmaps for protein-protein correlations using *heatmaply* package (version 1.5.0, https://talgalili.github.io/heatmaply/articles/heatmaply.html) [27]. All packages and versions used for visualisation are stated in the app.

## Results

### Frequent blood microsampling and pancreas pathology in CVB3-infected NOD mice

To study the effects of a type 1 diabetes associated enterovirus [4] on the circulating proteome, two independent longitudinal studies (denoted Study 1 and Study 2) were conducted. Female NOD mice, aged 8–9 weeks, were infected with CVB3 or mock infected. Previous studies have shown that the pancreas is permissive to CVBs, but infection of NOD mice at this age does not induce diabetes [19, 20]. For each mouse, 5 µl of blood was collected on individual filter paper discs before infection and at several time points thereafter until 14 days p.i. (Fig. 1a). DBS were stored at room temperature until analysis. In both studies infected mice lost weight from days 5–6 p.i. (ESM Fig. 1) and four mice were euthanised on days 9, 10 or 13 p.i. due to excessive weight loss (>15%; *n *= 2; ESM Fig. 1). Upon termination, buffer-treated mice had normal looking pancreas, while most infected animals had a smaller pancreas with clear histopathological signs of infection (ESM Fig. 2). Notably, no animals developed diabetes (data not shown).

### Proteome profiling of DBS samples collected from CVB3-infected NOD mice

DBS samples were analysed using PEA. Ninety-two proteins were measured in longitudinal DBS samples from all animals. The data were first normalised using Protein-specific Probabilistic Quotient Normalization (ProtPQN), as previously described [18] and no outliers were identified (ESM Fig. 3a). The data were then scaled and centred using *z* score transformation. After pre-processing, no significant differences in overall protein levels were found between the two studies (*p*>0.05), and the per cent variance explained by the first principal component (PC1) decreased from 80% to 17% (ESM Fig. 3b–c). Unless specified, the pre-processed and transformed data from the two studies were combined and used in downstream analyses.

To assess the reproducibility of the affinity proteomics method, we collected multiple concurrent samples (*n *= 6 in Study 1, *n *= 5 in Study 2) from the same animal. Using pre-processed data, we observed a mean CV of 10.2% and 20.5% across all proteins in Studies 1 and 2, respectively. Per sample, Spearman correlations of rho = 0.99 for Study 1 and rho = 0.94 for Study 2 were obtained for the inter-animal replicate samples (ESM Fig. 4) confirming the suitability of the data for further investigations.

The detectability of the proteomics data was calculated for each study using unprocessed normalised protein expression values by calculating the number of data points above the limit of detection (LOD). In Study 1, 64.2% of data points were above the LOD, compared with 61.1% in Study 2. This detectability did not differ significantly between the infected and control groups (ESM Fig. 5a, b). When divided by sampling day, in Study 2 a greater number of proteins were detected above the LOD in the infected group on day 3 p.i., whereas the detectability was lower on days 1, 9 and 14 p.i. (*p *< 0.05) (ESM Fig. 5d). No significant differences were found in Study 1 (ESM Fig. 5c).

### Longitudinal proteome profiling of DBS samples reveals dynamic and transient protein changes induced by CVB3 infection

To gain a first, unbiased overview of the temporal levels of circulating proteins, we performed hierarchical clustering of proteins over time and in response to CVB3 infection (Fig. 1b, c). The obtained protein clusters were then applied to the control data to illustrate the p.i. protein dynamics.

For example, in infected animals, proteins involved in immune responses such as CCL2, CXCL9, CXCL1, TNFRSF11B, LGMN and CCL5 increased after infection, peaking around day 4 p.i. Additionally, a noticeable yet minor increase in CCL2 levels was observed in the control group in the first 5–6 days p.i. (Fig. 1b, c).

Other proteins, including CCL20, GHRL and CLMP, increased in abundance in CVB3-infected mice from days 5–7 p.i. A cluster of proteins, including PARP1, RIOX2, FOXO1 and FLI1, decreased in level in infected mice between days 4 and 7 p.i. but returned to baseline by day 14. Conversely, protein levels for DCTN2, AXIN1, CNTN1 and CDH6 declined around day 5 p.i. and remained low until the end.

The control group displayed a more uniform distribution of *z* scores, with fewer changes in protein levels compared with infected mice. Some proteins, including YES1, remained unchanged over time in both groups (Fig. 1b, c). An interactive web interface was developed to enable browsing of protein-centric results (https://mouse-dbs-profiling.serve.scilifelab.se/). The interface presents the profile of each protein and summarises the data in terms of precision, variance and protein-protein correlations (ESM Fig. 6).

**Fig. 2:**
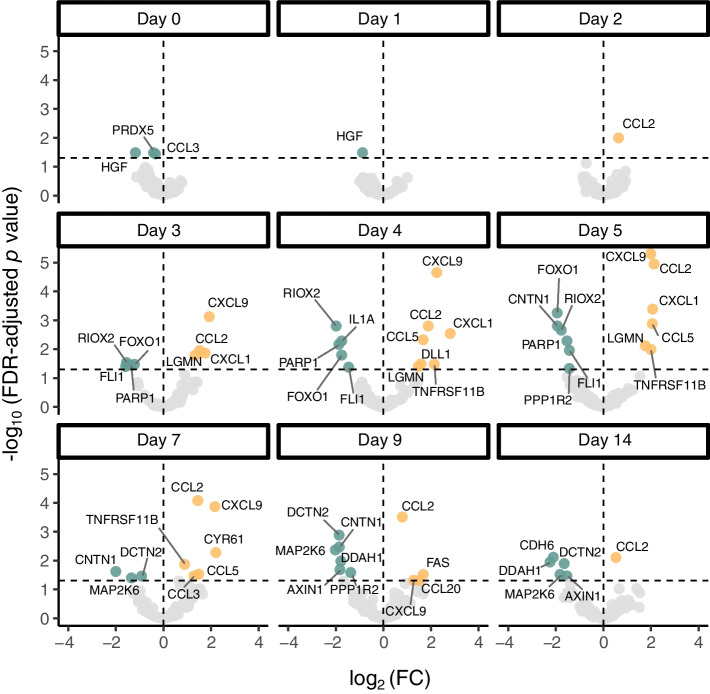
Protein dynamics across sampling time in CVB3-infected and control NOD mice. DBS samples were collected from CVB3- (*n *= 9) and mock-infected (*n *= 8) NOD mice for 14 days p.i. as described in Fig. 1a. Proteins were measured using PEA and the protein signals were transformed to *z* scores before combining the data from two independent studies. The figure shows volcano plots for each sampling day for the log_2_ (fold change) between the infected and control groups plotted against the FDR-adjusted *p* value obtained from two-sided Student’s *t* test. Each dot represents a protein. The horizontal dotted line represents a *p* value of 0.05, and proteins above are considered significant. The vertical dotted line represents a fold change of zero, and proteins to the right of the line are found at higher levels in the infected group and vice versa. Yellow and green dots are proteins that are up- or downregulated, respectively, in the infected mice compared with the controls. Grey dots are proteins that were not considered significantly different between infected and control animals. FC, fold change

Next, to identify infection-associated changes we performed statistical tests to compare the protein levels in infected mice with those in the control group at each time point (Fig. 2). Notably, CCL2 was significantly elevated in the infected group of animals compared with the uninfected controls as early as day 2 p.i. (FDR-adjusted *p* value <0.05), which persisted until day 14 p.i. (Fig. 2; ESM Table 1). Similarly, CXCL9 levels were elevated in the infection group from day 3 until day 9 p.i., indicating a temporal pattern in the host immune response. The highest number of proteins (*n *= 12) with significantly different time-dependent profiles between the infected and control animals were found on days 4 and 5 p.i. In the infected group seven proteins were elevated on day 4 p.i., and six were increased on day 5 p.i. By day 14 p.i., significant differences were detected in the levels of six proteins between the groups, with only CCL2 slightly elevated in infected mice compared with the controls (Fig. 2).

We also used GAMs to assess linear and non-linear relationships between protein levels and the number of days p.i. This revealed a notable number of non-linear relationships between time p.i. and protein levels for several proteins after Bonferroni correction (infected group *n *= 37, control group *n *= 8; Table 1; ESM Table 2). These included CCL2, CXCL9 and CXCL1, which fluctuated over time. Seven proteins (TNFRSF11B, CCL5, CCL2, CXCL9, CXCL1, GHRL and EDA2R) had significantly different time-detection profiles between the groups using this model.

**Table 1:** *p* values from GAM for top ten proteins differentiating CVB3-infected and control animals

Gene	UniProt	*p* value for infection status	Deviance explained by model
*Ccl2*	P10148	2.86 × 10^−8^	0.89
*Cxcl9*	P18340	3.56 × 10^−6^	0.81
*Ccl5*	P30882	0.00059	0.74
*Cxcl1*	P12850	0.0062	0.75
*Ghrl*	Q9EQX0	0.0086	0.56
*Eda2r*	Q8BX35	0.011	0.71
*Tnfrsf11b*	O08712	0.017	0.69
*Dll1*	Q61483	0.076	0.32
*Foxo1*	Q9R1E0	0.15	0.59
*Riox2*	Q8CD15	0.36	0.47

### DBS protein levels predict infection status early after infection

**Fig. 3:**
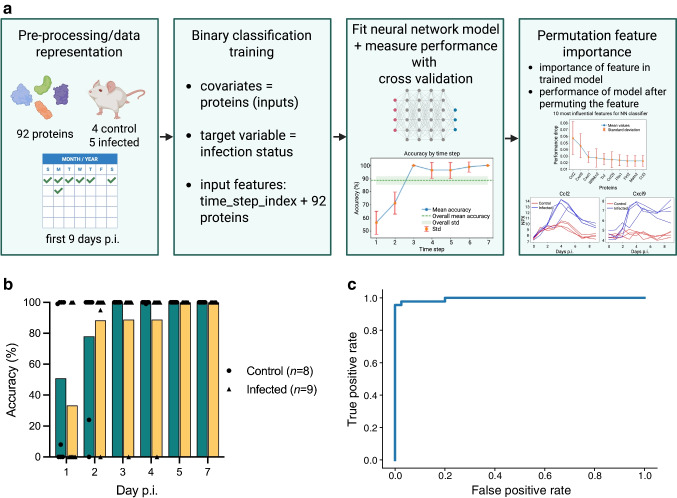
Model performance for classifier to predict infection status. (**a**) ML pipeline for preliminary analysis of Study 1 samples aimed at identifying key features for infection prediction. Permutation feature importance was applied to quantify the contribution of covariates (proteins) to the model’s output. Here the model is a binary classifier that takes all 92 protein measurements at each time step as inputs for predicting infection status. Feature importance is assessed by randomly permuting each feature’s values and measuring the resulting drop in performance compared with the original unaltered input. Notably, permuting CCL2 and CXCL9 resulted in the largest accuracy drops, underscoring their significance. Their predictive relevance is visually confirmed in the plot depicting their temporal evolution. Note that the model used in the preliminary analysis differs from the one employed in the main experiments. In the ‘Fit neural network’ box, step 1 = day 1, step 2 = day 2, step 3 = day 3, step 4 = day 4, step 5 = day 5, step 6 = day 7 and step 7 = day 9. Created in BioRender. Byvald, F. (2024) https://BioRender.com/y07z021. (**b**, **c**) Results from the model used in the main analysis including data from both independent studies (Study 1 and Study 2). (**b**) Percentage accuracy of the model up to day 7 days p.i. in control (*n *= 8; green; circles) and infected (*n *= 9; yellow; triangles) mice. Individual animals are represented by individual symbols. (**c**) ROC curve illustrating the performance of the binary classification for infection status for the validation set (Study 2). Sensitivity (true positive rate) is plotted against 1-specificity (false positive rate). The AUROC was 0.995. NN, neural network; Std, standard

By applying ML techniques to our longitudinal proteome dataset (Fig. 3a), we next constructed a classifier for infection prediction. Preliminary analyses using the dataset generated in Study 1 and application of permutation feature importance identified CCL2 and CXCL9 as the most informative biomarkers for this task. Using an MLP classifier, we leveraged the trajectories of these proteins from day 0 to various time points p.i. By day 2 p.i., we accurately predicted the infection status for 13 out of 17 mice (76%) and by day 3 p.i., 16 out of 17 (94%) (Fig. 3b and ESM Table 3). For the remaining mouse, which exhibited a delayed response trajectory, infection status was correctly predicted by day 4 p.i. Overall, after day 2 p.i., our classifier correctly predicted the infection status for 83 out of 85 mouse-timestep combinations, equating to an accuracy rate of approximately 97.6%. A receiver operating characteristic (ROC) curve was calculated for the binary classification of infection status (Fig. 3c), and the area under the ROC curve (AUROC) was 0.995. A ROC for the training set is shown in ESM Fig. 7.

### Monitoring-informed intervention prevents virus-induced diabetes development

The ML-based classifier demonstrated that infection status could be predicted early p.i. (day 2), even before clear signs of infection, like weight loss, became noticeable (ESM Fig. 1). Therefore, we next explored whether this early disease prediction could offer a window for preventive treatments.

For this we employed an experimental model for CVB-induced type 1 diabetes, the SOCS-1-tg mouse [19–21]. In these NOD mice, the overexpression of the human *SOCS1* gene under the control of the human insulin promoter renders the beta cells unresponsive to IFN stimulation. Consequently, in the beta cells, critical autonomous antiviral defence mechanisms which are typically activated during virus infections via type I IFNs are compromised. Therefore, the beta cells succumb to CVB infection, and SOCS-1-tg mice develop diabetes approximately 5–12 days after infection [19–21].

**Fig. 4:**
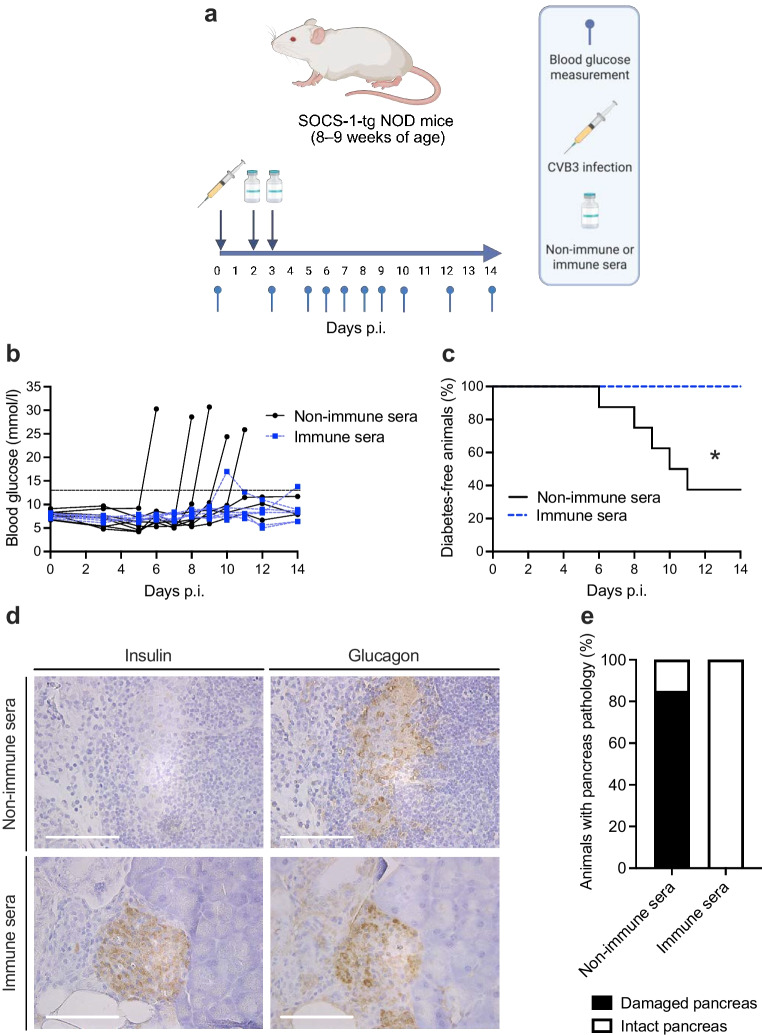
Early intervention prevents virus-induced type 1 diabetes. SOCS-1-tg mice were infected with CVB3 (10^5^ PFU CVB3, i.p.). On days 2 and 3 p.i., animals were treated with either non-immune (*n *= 8) or immune sera (*n *= 6) by i.p. injection (total volume 200 µl/mouse). (**a**) Experimental schematic. Created in BioRender. Byvald, F. (2024) https://BioRender.com/b43t411. (**b**) Blood glucose values of individual animals treated with non-immune sera (*n *= 8; black line) or immune sera (*n *= 6; dotted blue line). Mice were deemed diabetic when the blood glucose level was equal to or exceeded 18 mmol/l, or when two consecutive daily measurements ranged between 13 and 18 mmol/l. Dotted black line marks 13 mmol/l glucose. (**c**) Diabetes incidence curves summarising results shown in (**b**). *p<0.05 comparing the two groups by logrank (Mantel–Cox) test. (**d**) Representative images of sequential pancreas sections from mice infected with CVB3 and treated with non-immune or immune sera. Pancreas was stained for insulin (panels on left) or glucagon (panels on right). Positive areas are stained brown. Scale bars, 100 µm. (**e**) Percentage of animals with damaged and intact tissue morphology in pancreas specimens from mice treated with non-immune sera (*n *= 7) or immune sera (*n *= 5). The infection status of two animals, one from each treatment group, could not be determined histologically due to insufficient quality of the formalin-fixed, paraffin-embedded sections, and were excluded from the assessment

While potent antiviral treatments for CVB infections are lacking, passive immunisations have shown promise [28–30]. We explored this and generated non-immune and immune sera from separate groups of NOD mice. Subsequently, SOCS-1-tg mice were infected with CVB3, and treated with non-immune or immune sera on days 2 and 3 p.i. (see Fig. 4a). In the non-immune sera-treated group, diabetes was triggered in five out of eight mice (Fig. 4b, c). Histological assessment of pancreas revealed that most of these mice had extensive exocrine tissue damage and minimal or absent insulin-positive cells in the islets (Fig. 4d, e, ESM Fig. 8). Remarkably all six mice treated with immune sera remained diabetes free (6/6) (Fig. 4b, c) with intact pancreases and islets expressing insulin and glucagon (Fig. 4d, e, ESM Fig. 8).

## Discussion

To achieve preventive treatments for people at risk of developing type 1 diabetes through precision medicine it is crucial that disease-triggering events that occur in the early, non-symptomatic stage(s) are identified. While sensors and apps can help track symptoms, conditions and/or behaviour, we still lack the tools to monitor molecular changes that are indicative of early disease. Of the accessible sample types, blood provides system-wide and organ-specific insights into health. Proteins, lipids and other small molecules can be reliably measured in large numbers to track changes [31]. However, the timing of blood draws is crucial for identifying changes before disease tipping points are reached, thereby necessitating longitudinal sampling in presymptomatic individuals.

Thanks to the development of self-sampling DBS methods, new routines for health monitoring have been introduced [32]. The ease of fingertip blood collection and the advent of technologies that are compatible with small sample volumes raises the question of whether data collected after frequent sampling can effectively track disease progression and facilitate early preventive interventions. Using type 1 diabetes models combined with the identification of proteins in DBS, we asked if frequent blood microsampling and the analysis of time-resolved protein patterns could identify predictive disease markers and the optimal time window for effective disease interventions. We demonstrate that repeated sampling of small volumes of blood (5 µl) followed by multiplexed protein analysis of DBS samples can efficiently monitor proteome changes during an infection with a type 1 diabetes associated virus and in turn enable timely disease interventions.

Through analysis of DBS samples using a commercially available protein panel, we monitored >90 proteins relevant to various biological processes and disease areas. These included immune response proteins, growth factors, cell signalling proteins, proteins involved in cardiovascular and neurological diseases and various cancer associated proteins. Among the immune response proteins, the chemokine CCL2 showed a transient increase in infected and control animals. It peaked in CVB3-infected animals around day 5 p.i., while the increase was earlier and lower in the controls. This suggests the initial blood sampling triggered CCL2 production, which concurs with the known crucial role of CCL2 in the recruitment of monocytes and other immune cells to sites of inflammation caused by tissue injury or infection [33]. Minor but similar temporary increases in other immune-related proteins, including CCL20 and IL1β, were detected in both groups (Fig. 1). This important observation demonstrates that the sampling procedure itself, especially when samples are repeatedly taken from the same location, may temporarily impact certain protein levels. The protein data from the uninfected control group, however, displayed a more uniform distribution of *z* scores with fewer protein changes than the infected group (Fig. 1). This indicates that, overall, the repeated handling and blood sampling was well tolerated.

Recent work summarised the current understanding of immune responses to CVB infection [4]. Our study offers high resolution of systemic proteome alterations that occur during the acute phase of an infection. The greatest differences in circulating protein levels were observed between days 4 and 7 p.i. Despite under-representation in the panel, most of these proteins are associated with the immune system. Given that robust innate antiviral defence mechanisms are activated and peak earlier than day 4 p.i. [29, 34], future studies using a panel that covers additional immune-related proteins may identify further candidates with altered abundance early after infection. Moreover, unique patterns may be found which could distinguish enterovirus infections from those with other viruses. Complementary insights into infection biology could also be provided by other proteomics methods (e.g. MS), alongside the analysis of other molecules including metabolites and/or lipids. Our study underscores the dynamic nature of protein changes in response to acute infection, with specific proteins exhibiting significant temporal expression patterns. Importantly, these patterns would have been missed with less frequent sampling.

The dynamic protein changes observed during acute CVB infection also revealed proteins that decreased in abundance. For example, FOXO1 and CNTN1 levels temporarily dropped during infection. The implications of these changes are an area of future interest.

Interestingly, we correlated altered protein expression with a physical readout, highlighting an indirect infection effect. In CVB3-infected mice, there was a late increase in ghrelin levels which significantly correlated with weight loss (*r = *−0.51;* p *= 2.1 × 10^–6^; as determined by Spearman correlation). Ghrelin, known for stimulating appetite and regulating hunger and energy balance, typically increases after significant weight loss [35]. The CVB3-infected animals experienced weight loss throughout the infection, which was likely due to their extensive exocrine pancreas damage. Collectively, this highlights that through the analysis of longitudinal DBS samples, we can identify molecular footprints induced by CVB3 infections that are indicative of the direct, indirect and broader impacts that CVBs have on health.

ML methods are becoming pivotal for analysing complex datasets, particularly for the prediction and staging of diseases, and for individualised patient care [10]. After applying ML techniques to our longitudinal proteome dataset, we successfully constructed a classifier for infection prediction. The proteins CCL2 and CXCL9 emerged as the most informative biomarkers for this task, consistent with classical approaches. By day 2 p.i. we predicted the infection status with an accuracy rate of >75% which was >95% by day 3 p.i. The effectiveness of this prediction for informing relevant time points for treatment interventions was demonstrated using an experimental model for virus-induced type 1 diabetes [19–21]. Infected animals treated with immune sera on days 2 and 3 p.i. were protected from significant pancreatic damage and type 1 diabetes development. This underscores the potential of ML-based approaches in using plasma proteomics for early and precise infection status prediction, opening new avenues for appropriate interventions and improved disease management.

Prospective cohort studies which have followed children from infancy have significantly enhanced our understanding of type 1 diabetes pathogenesis, including the sequence of islet AAb appearance [36–39]. However, these studies have not yet identified the early triggers and the precise time when beta cell autoimmunity is induced, which is crucial for implementing preventive therapies. This limitation may be partly due to the relatively lengthy sampling intervals (e.g. 3 months or more between samples). More frequent blood sampling may have facilitated the detection of a disease-triggering event, such as infection by a virus with known islet-tropism.

This study presents a monitoring scheme for frequent blood microsampling which is translatable to humans. It addresses several current bottlenecks in identifying transient molecular indicators of disease triggers in islet autoimmunity and type 1 diabetes progression. First, many screening initiatives still collect samples at primary care centres, which is impractical for participants and limits sampling frequency. Additionally, current initiatives for at-home capillary blood sampling for islet AAb screening require up to 250 µl blood [40], posing a challenge that may lower compliance. Previously we reported successful unsupervised volumetric DBS sampling by finger-pricking in a home setting [17], which facilitated frequent sample collection. Here, we extended these efforts by showing that as little as 5 µl of whole blood is needed to reveal blood proteome signatures, significantly simplifying the collection procedure and reducing the risk of insufficient sample volume. Furthermore, we report the successful proteome analysis of DBS samples stored at room temperature, which simplifies at-home storage, lowers the risk of compromising sample integrity during transport and reduces pre-analysis storage costs in diagnostic or clinical laboratories. While daily DBS sampling may not be possible in humans, a weekly or bi-weekly collection regimen could be feasible.

Our study has some limitations. First, it was conducted in a mouse model rather than in humans. However, our aforementioned work demonstrated the feasibility of performing DBS sampling followed by proteomics analysis in human participants [17]. Second, while current PEA-based assays can monitor 5000–6000 human proteins, the available assays for murine samples are more limited. Consequently, applying a similar monitoring approach in humans would provide a broader more comprehensive insight into proteome changes induced by infection or another environmental factor.

In conclusion, our proof-of-concept study highlights the importance of more frequent sampling in capturing temporal protein changes induced by a disease trigger. This new approach for analysing frequently collected DBS samples provides a more accurate picture of biomarker fluctuations and holds significant potential for identifying novel disease-predictive molecular markers and informing early intervention strategies for type 1 diabetes. Moreover, it may facilitate the discovery of disease-specific endotypes. Integrating genetic risk scores with measurements of AAbs or other markers with a proteome-informed intervention strategy could significantly expedite the prevention of type 1 diabetes. Additionally, incorporating repeated sampling and proteomics analysis in clinical trials is an attractive opportunity to better understand the trajectories of treatment responses.

## Supplementary Information

Below is the link to the electronic supplementary material.

ESM (PDF 8.05 MB)

ESM Tables (XLSX 68 KB)

## Data Availability

Upon publication, all data needed to evaluate the conclusions in the paper are present in the paper, the supplementary materials, and/or are accessible at https://doi.org/10.17044/scilifelab.27368322. Additional visualisations are presented in the Shiny app interface (https://mouse-dbs-profiling.serve.scilifelab.se/). Material requests should be sent to the corresponding authors M. Flodström-Tullberg and J. M. Schwenk.

## Abbreviations

AAbs: Autoantibodies
AUROC: Area under the ROC curve
CVB: Coxsackievirus B
DBS: Dried blood spots
FDR: False discovery rate
GAMs: Generalised additive models
LOD: Limit of detection
ML: Machine learning
MLP: Multi-layer perceptron
PEA: Proximity extension assay
PFU: Plaque forming units
p.i.: Post infection
ROC: Receiver operating characteristic
SOCS-1-tg: *SOCS1* transgenic

## Code availability

Codes used in this work will be made available upon publication via the Schwenk Lab’s GitHub account under ‘Mouse-DBS-Profiling’.

## Authors’ relationships and activities

MFT has served on the scientific advisory board of Provention Bio Inc. (acquired by Sanofi in 2023). NR is a co-founder and shareholder of the microsampling companies Capitainer AB and Samplimy Medical AB, and an inventor of several patents on microsampling solutions. Unrelated to this work, JMS has received travel support from Olink AB, and via the institution, conducted contract research for Capitainer AB. All other authors declare that there are no relationships or activities that might bias, or be perceived to bias, their work.
