# Supplementary material for: Frequent longitudinal blood microsampling and proteome monitoring identify disease markers and enable timely intervention in a mouse model of type 1 diabetes
Source: Diabetologia. 2025 Aug 4;68(10):2277–89. doi: 10.1007/s00125-025-06502-7 (PMC12423192; doi:10.1007/s00125-025-06502-7)
Supplement: Supplementary file 1 — ESM (PDF 8.05 MB) [file 125_2025_6502_MOESM1_ESM.pdf]

ESM for

Frequent longitudinal blood microsampling and proteome monitoring identify disease markers and enable timely intervention in a mouse model of type 1 diabetes

Anirudra Parajuli *et al*.

*Corresponding authors. Email: malin.flodstrom-tullberg@ki.se and jochen.schwenk@scilifelab.se

This file includes:

ESM Methods

ESM Figures

ESM Fig. 1 Percentage weight change in mock and CVB3-infected NOD mice.
ESM Fig. 2 NOD mouse pancreas displays clear signs of pathology after CVB3 infection.
ESM Fig. 3 Initial data analysis of proteomics data generated from DBS samples collected from CVB3- or mock infected mice.
ESM Fig. 4 Reproducibility analysis for DBS sampling and proteomics analysis.
ESM Fig. 5 Protein detectability analysis in proteomics assays used to analyse DBS samples from CVB3 infected and mock infected animals.
ESM Fig. 6 An overview of an interactive web-based interface for proteomics data generated through the analysis of longitudinally collected DBS samples.
ESM Fig. 7 ROC curve for the training set.
ESM Fig. 8 Pancreatic beta cell loss and exocrine pancreas damage in CVB3 infected SOCS-1-tg mice is prevented by an early intervention.

ESM References

ESM Tables (separate file)

ESM Methods

Animal husbandry and monitoring of animal health

NOD and *SOCS1* transgenic (SOCS-1-tg) NOD [1, 2] mice were bred and maintained under specific pathogen free conditions at the Karolinska University Hospital Huddinge, Stockholm, Sweden. All experiments received approval from a local ethics committee (Linköpings djurförsöksetiska nämnd, Dnr 9222-2019 and 04291-2024) and were conducted in line with the NIH principles of Laboratory Animal Care, institutional guidelines at Karolinska Institutet and Swedish national laws. The animals were kept in ventilated cages with unrestricted access to food and water. Each cage housed a maximum of 5 mice. No animals were single housed. Mice were randomly allocated to different treatment groups. Comprehensive health checks were performed and changes in health status monitored (including weight fluctuations, changes in natural behaviour, porphyria, movement and posture, piloerection, respiration, and skin condition). The researchers were aware of the experimental groups throughout the experiments. At the end of the experiment, at a terminal time point deemed by weight loss (>15% of the maximum body weight during the experimental duration) or at the onset of diabetes, the mice were anesthetised with isoflurane, and then euthanised by cervical dislocation.

Blood collection, glucose measuring, and monitoring of diabetes development

Blood was collected for proteome analysis from the tip of the tail. Prior to each collection the tail was wiped with 70% ethanol. On the first collection time point, a small portion (<1 mm) of the tail extremity was removed using sterile scissors. A small droplet of blood (less than 10 µl) was extracted by gently holding the mouse by its tail. From this, 5 µl was pipetted onto a paper-based sampling disc (Capitainer AB, 710-0020, Solna, Sweden) and left to dry until analysis. The scab was gently removed for subsequent blood collections. Blood glucose levels were measured using a Bayer Contour XT blood glucose meter (Bayer, Basel, Switzerland) in tail vein blood samples. Diabetes was diagnosed when blood glucose levels were equal to or exceeded 18 mmol/l or when two consecutive daily measurements ranged between 13 and 18 mmol/l.

Histology and immunohistochemistry

Pancreas specimens were fixed in formalin, embedded in paraffin and cut into 5 µm thick sections with a Microm HM355S microtome (Thermo Scientific, Kalamazoo, MI, USA). Pancreas tissue morphology was visually assessed in tissue stained with hematoxylin-eosin (H&E; Haemotoxylin, Histolab, #01820, Västra Frölunda, Sweden; Eosin, Sigma-Aldrich, HT110232, Bohos, Sweden). Pancreas was considered damaged when intact acini of the exocrine tissue were scarcely found or absent, resulting in void space between islets and ductal cells, causing loss of tissue integrity. Immunohistochemistry for insulin and glucagon was carried out as previously described [1, 3] using guinea pig anti-insulin (N1542, DakoCytomation. Glostrup, Denmark; 1:20,000 dilution) and rabbit anti-glucagon (ab92517, Abcam. Cambridge, UK; 1:24,000 dilution) antibodies and with biotinylated anti-guinea pig (for insulin; DakoCytomation; 1:200 dilution) and biotinylated polyclonal anti-rabbit (for glucagon; Dako, Glostrup, Denmark; 1:500 dilution). The antibodies were validated in unrelated mouse pancreas. Sections were deparaffinised, rehydrated and then treated with 0.2M glycine for 30 minutes. They were washed twice in PBS and were then blocked in 10% NGS (Dako, X0907, Agilent, CA, USA) in PBS for 30 minutes prior to addition of the primary antibodies diluted in 2% NGS (in PBS) overnight at 4°C. The sections were washed 3x3 minutes in PBS and then secondary antibodies diluted in 2% NGS were added for 1 hour at room temperature. After washing 3x3 minutes in PBS, the sections were blocked in 1% hydrogen peroxide/methanol for 15 minutes, washed for a further 3x3 minutes in PBS and then ABC kit (Vectastain, PK-4000, Vector Laboratories, CA, USA) was added according to the manufacturers instructions. Insulin and glucagon positivity were revealed by DAB staining according to the kit protocol (DAB Substrate Kit, SK-4100, Vector Laboratories), sections were washed quickly 4 times in PBS and then counterstained using hematoxylin (Histolab, #01820, Västra Frölunda, Sweden) for 1 minute. Sections were washed in dH_2O until the water ran clear then incubated in 2% $NaHCO_3$ for 30 seconds, followed by a final quick wash in PBS. The sections were dehydrated and coverslips were mounted in Pertex mount (Histolab, #00840, Askim, Sweden). Stained tissue

was visually examined, and images were acquired using a Leica DFC495 light microscope (Leica Microsystems Ltd. Heerbrugg, Germany) and LAS X software (v. 3.0.14.23224; Leica Microsystems Ltd.).

Blood sample processing and proteomics assay

Filter discs with dried blood were randomised (using the Microsoft Excel randomisation function) in flat-bottom 96-well plates (VWR, #10861-562, Sweden) and eluted in 50 µl PBS with 0.05% Tween20 (Thermo Fisher Scientific) and protease inhibitor (cOmplete Tablets EASYpack, Roche, #04693116001, Solna, Sweden). After 1 h incubation with gentle shaking (170 rpm) at 23°C, the plates were spun at 400 g (Allegra X-12R, Beckman Coulter Inc, Solna, Sweden) for 1 minute to remove any liquid from the plate seal. The supernatant was then transferred to a 96-well PCR plate and centrifuged at 2100 g (Allegra X-12R, Beckman Coulter Inc.) for 3 min. After centrifugation, 40 µl of the supernatant was transferred to a new 96-well PCR plate before starting the proteomics assay.

The Olink Target 96 Mouse Exploratory panel (Art # 95380; Batch B24933 for Study 1, Batch B30620 for Study 2, Olink, Uppsala, Sweden) was used to measure 92 circulating proteins in the DBS eluates according to the manufacturer's protocol for plasma samples. Briefly, 1 µl of each sample was incubated overnight with oligonucleotide-coupled antibody pairs, which only allow the hybridization of the DNA strands if the antibodies bind to a common protein in close proximity. In a subsequent step, proximity extension was used to create unique DNA reporter sequences for each protein and control, which were amplified and detected by real-time PCR using a Biomark™ HD (Fluidigm, AH diagnositics, Solna, Sweden). The two sample batches were processed separately.

The Ct values were transformed into Normalised Protein eXpression (NPX) values using Olink NPX Signature (v. 1.6.0.0 for Study 1, v1.9.0 for Study 2). NPX is Olink's arbitrary unit of relative protein levels and is reported on the log2 scale. The data sets of Study 1 and Study 2 were independently normalised per protein using ProtPQN to reduce sample-to-sample variation in the DBS [4, 5]. The presence of sample outliers was investigated by plotting the sample median against the interquartile range (IQR). Samples with a standard deviation (SD) of 5 or more were flagged as outliers. The data was then scaled and centered by transforming it to *z* scores per protein and per study.

Statistical analysis

The reproducibility of the DBS sampling and the proteomics assay process was tested in biological replicates taken at the same timepoint (six samples from one mouse in Study 1, and five samples from one mouse in Study 2), and in technical duplicates. Spearman correlations between replicates were determined using the corr.test function from *psych* package (version 2.4.6.26; https://rdocumentation.org/packages/psych/versions/2.5.3) and visualised with *ggpairs* functions from the *GGally* package (version 2.2.1; https://ggobi.github.io/ggally/). The compatibility of the transformed data from the two studies was assessed using Wilcoxon rank sum test using the *wilcox.test* function from the *stats* R-package, and visualisation using principal component analysis (PCA) plots. Heatmaps for the average protein expression for the infected and control groups per sampling day were generated using the *ComplexHeatmap* package in R (version 2.20.0; https://bioconductor.org/packages/ComplexHeatmap/). Proteins were clustered using the Euclidean distance matrix. Differences in detectability between infected and uninfected groups were investigated using Fisher's exact test using the *fisher.test* function from the stats package. Two sample Student's T-tests were used to determine differences in protein levels between infected and control mice at each time point.

Generalised Additive Models (GAMs) were used to assess the relationship between protein expression levels and infection status over time. The analysis used the *mgcv* package in R (version 1.9-1; https://cran.r-project.org/web/packages/mgcv/index.html). A separate GAM was fitted for each protein assay, where the *z* score was considered a function of infection status and sampling day with a smoothing term for sampling day with separate smooths for each infection status (Table 1, ESM Table 2). The smoothness parameter (k) was set to 6 to control for potential overfitting. Repeated measures within subjects were accounted for by adjusting for subject-dependent variations in intercepts. The models were fitted using the Restricted Maximum Likelihood

method to optimise the smoothing parameter. For *p* values below $2x10^{-16}$, the values were substituted with $2x10^{-16}$ due to limitations in machine epsilon accuracy, as indicated in ESM Table 2.

Generation of a classifier to predict infection status

ML techniques were employed in python (version 3.7) to develop a binary classifier aimed at predicting the infection status of animals followed in the longitudinal study. Specifically, the classifier utilised the protein trajectories of each mouse from Day 0 until a given time step T to make its predictions. The two most informative proteins for this task, identified through preliminary analyses, were CCL2 and CXCL9, and their measurements served as the primary inputs for the model.

Each protein's trajectory from Day 0 to Day T was encoded into a 4-tuple consisting of the following features: Timestep T

- Standard deviation of protein measurements from Day 0 until Day T
- Range length of protein measurements from Day 0 until Day T
- Total variation of protein measurements from Day 0 until Day T

Where:

- Range length was defined as the difference between the maximum and minimum values of protein measurements from Day 0 to Day T.
- Total variation was the sum of the absolute values of the differences between consecutive measurements from Day 0 to Day T.

Thus, for each mouse, M, at timestep T, the representation is encapsulated in a 7-tuple: (T,std_devCcl2,range_lengthCcl2,total_variationCcl2,std_devCxcl9,range_lengthCxcl9,total_variationCxcl9). We chose a Multi-Layer Perceptron for our classifier due to its simplicity and effectiveness in binary classification tasks [6, 7]. The dataset comprised of 17 mice, each measured at 7 distinct time points in addition to Day 0, resulting in a total of 119 data points (17 mice × 7 time steps). A leave-one-out cross-validation approach was employed to validate the model. In this procedure, we iteratively selected one mouse for testing while training the classifier on the remaining 16 mice. This process was repeated 17 times, ensuring that each mouse was used as a test case once. To further ensure the robustness of our results, this cross-validation process was repeated 100 times.

A ROC curve for the binary classification of infection status was generated using the 17x5 output scores taken from the 17 classifiers trained with the leave-one-out cross-validation approach. Each classifier provided five predictions for the days following day 2 (i.e., days 3, 4, 5, 7, and 9).

ESM Figures

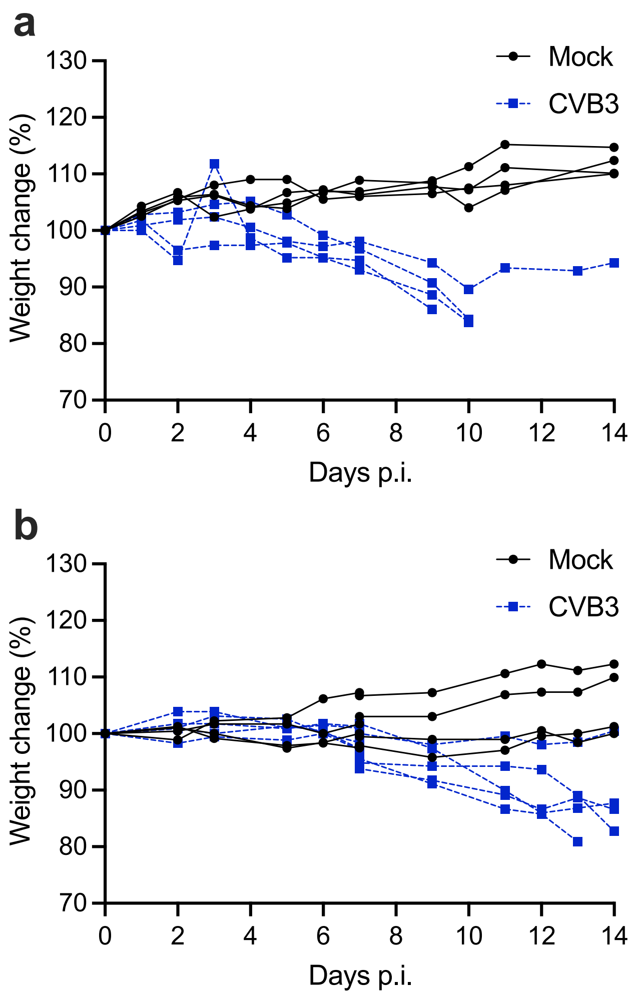

ESM Fig. 1 Percentage weight change in mock and CVB3-infected NOD mice. (**a** and **b**) NOD mice aged 8-9 weeks were infected with CVB3 (200 µl RPMI medium containing 10^5 PFU CVB3, i.p.) or mock infected (200 µl RPMI medium, i.p.). Weight measurements were taken as indicated, and animals were euthanised on day 14 p.i. or when weight loss exceeded 15 % of their maximal weights. The results from two separate studies are shown (a, Study 1 and b, Study 2). Each line represents an individual animal (black lines: mock-infected animals; hatched blue lines: CVB3-infected animals). In panel a: mock-infected animals, *n* = 4; CVB3-infected animals, *n* = 4. In panel b: mock-infected animals, *n* = 4; CVB3-infected animals, *n* = 5.

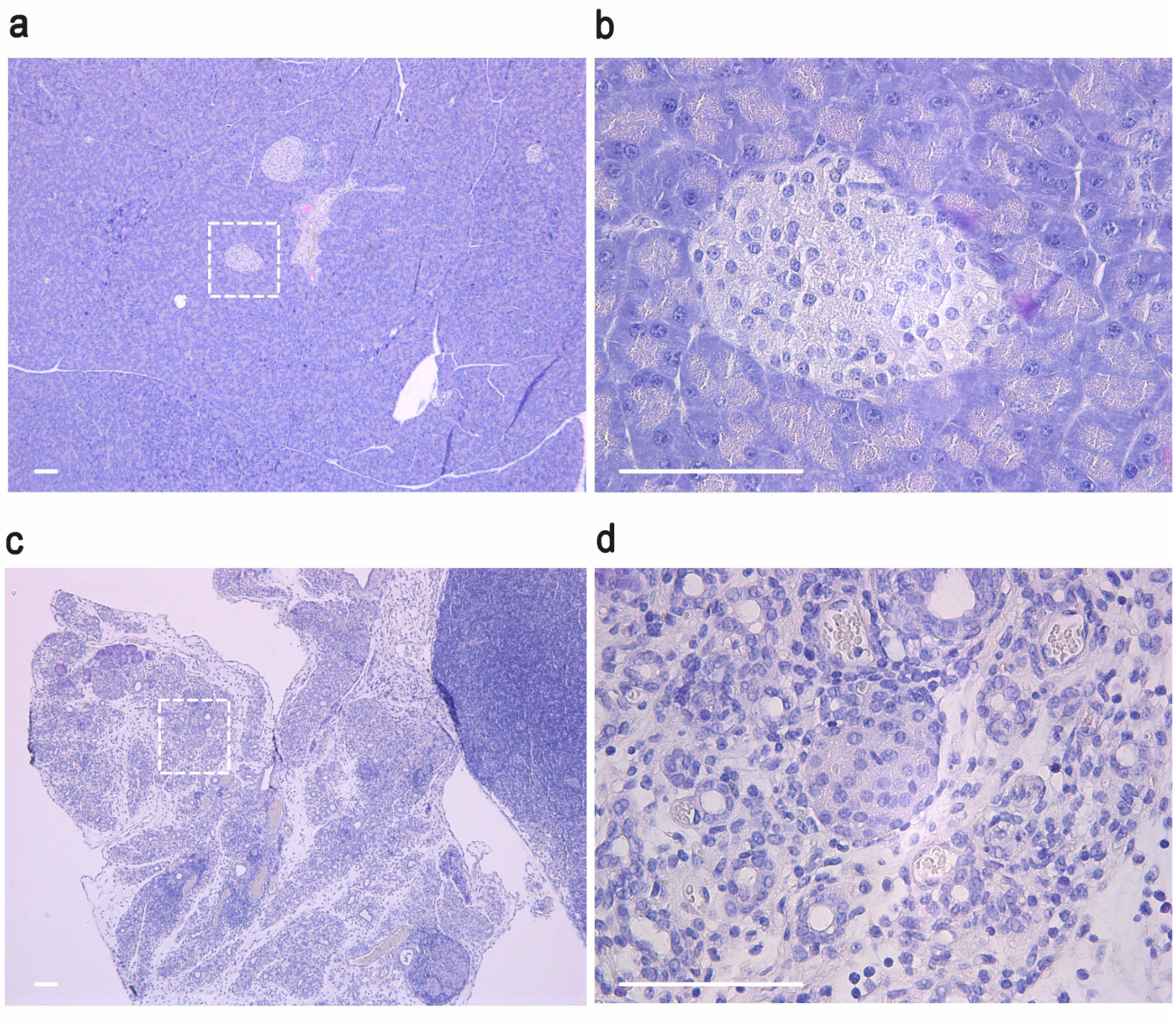

ESM Fig. 2 NOD mouse pancreas displays clear signs of pathology after CVB3 infection. NOD mice aged 8-9 weeks were infected with CVB3 (200 µl RPMI medium containing 10^5 PFU CVB3, i.p.) or mock infected (200 µl of RPMI medium, i.p.). Animals were sacrificed on day 14 p.i. or if their weight loss exceeded >15% of their highest body weight. (**a** and **b**) Representative images of a H&E stained formalin fixed pancreas specimen from a mouse that was mock-infected and sacrificed on day 14 p.i. (**c** and **d**) Representative images of a H&E stained formalin fixed pancreas specimen from a mouse that was infected with CVB3 and sacrificed on day 14 p.i. Images taken at 5x magnification (a and c) and at 40x magnification (b and d). Scale bars = 100 µm.

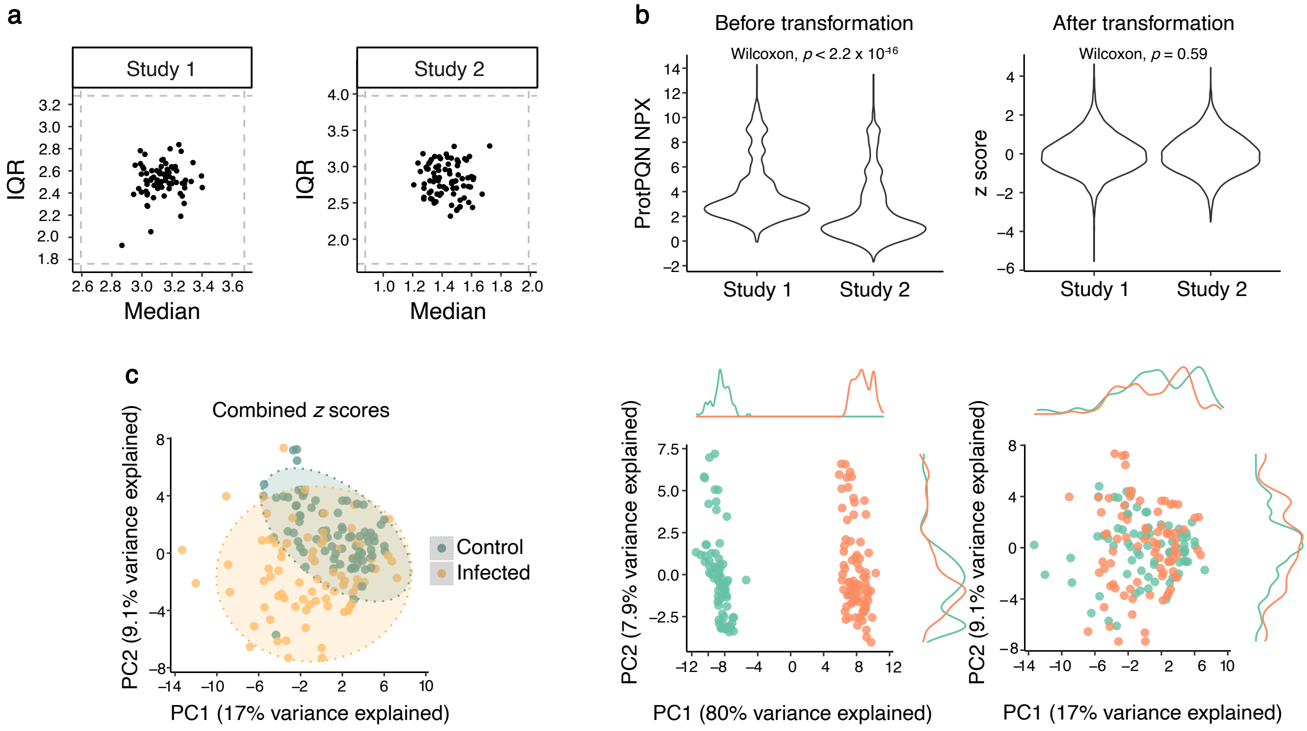

ESM Fig. 3 Initial data analysis of proteomics data generated from DBS samples collected from CVB3- or mock infected mice. DBS samples were collected from CVB3- or mock-infected NOD mice over 14 days p.i. in two separate studies (Study 1 and Study 2) as outlined in Fig. 1a. Proteins were measured in the samples using PEA. (**a**) Outlier analysis: Sample median against sample interquartile range (IQR) for each study. Each point is a sample, and the dotted line represents a threshold of 5 standard deviations from the mean IQR or sample median. Samples outside the threshold may be outlier samples. Here, no samples are outside of the threshold. (**b**) Data processing: Effect of *z* score transformation on the Olink data from the two studies. Overall protein signals (upper panel) for the two studies were significantly different before transformation using Wilcoxon Rank sum test, but not after. The lower panel shows overlapping datasets after transformation. (**c**) Data overview: principle analysis component plot showing the separation between infected and control group for *z* transformed data. PC, principle component.

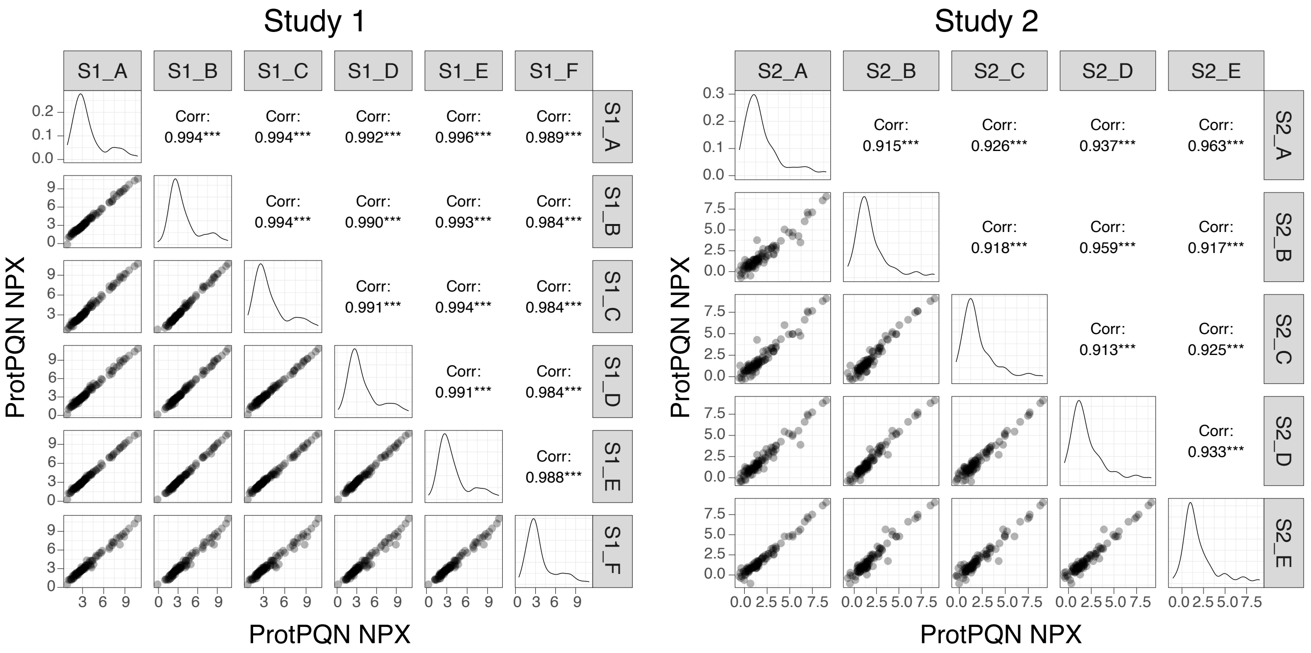

ESM Fig. 4 Reproducibility analysis for DBS sampling and proteomics analysis. In two separate studies (Study 1 and Study 2), DBS samples were collected from CVB3- and mock-infected NOD mice over 14 days as indicated in Fig. 1a. At the end of each study, multiple DBS samples were collected from a single mock-infected animal. Proteins in the DBS replicate samples from both studies were measured via PEAs. Shown are Spearman correlations for the DBS samples taken within each study (right panel, Study 1, *n* = 6; left, Study 2, *n* = 5).

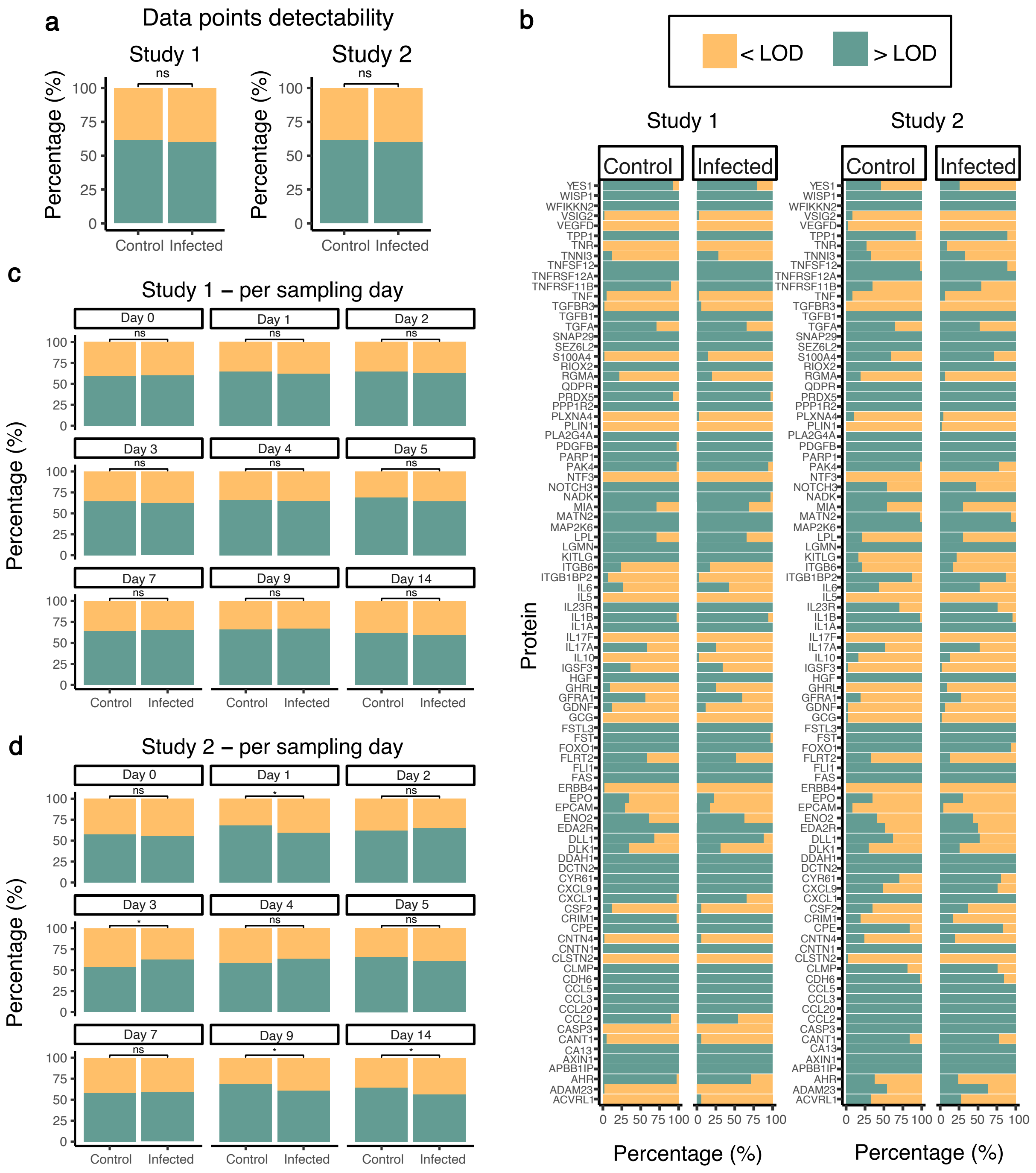

ESM Fig. 5 Protein detectability analysis in proteomics assays used to analyse DBS samples from CVB3-infected and mock-infected animals. DBS samples were collected from CVB3- or mock-infected NOD mice over 14 days after infection in two separate studies (Study 1 and Study 2) as indicated in Fig. 1a and proteins were measured in these samples via PEA (a total of unique 92 proteins were included). The obtained protein signal for each sample was compared to the limit of detection (LOD) for each protein. (**a**) Percentage of data points above and below the LOD in infected and control group for Study 1 (left) and Study 2 (right). Green indicates the data points above LOD, and yellow the datapoints below the LOD. The differences between the groups were not significant (ns) in either of the studies. (**b**) Detectability per protein and per group for the two studies. (**c** and **d**) Detectability per group and per sampling day for Study 1 and 2, respectively. Significance between groups were calculated using Fisher's Exact test. * $p < 0.05$.

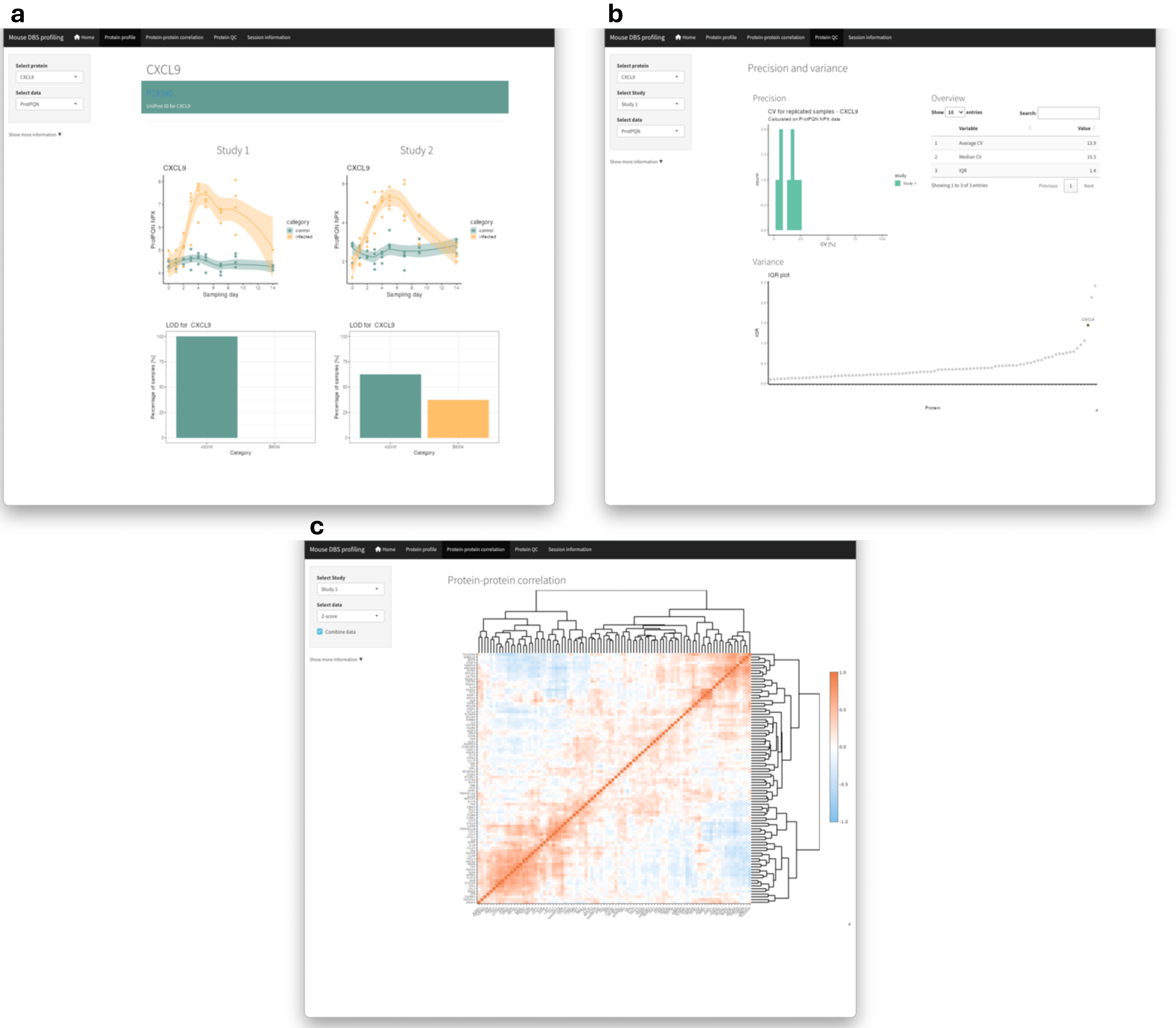

ESM Fig. 6 An overview of an interactive web-based interface for proteomics data generated through the analysis of longitudinally collected DBS samples. An interactive web-based interface was developed that contains the proteomics data measured in DBS samples from CVB3- and mock-infected mice. (**a**) The app has a tab showing the protein profiles and detectability for each protein included in the study. (**b**) A second tab shows the CVs and inter-quartile-range for each protein. (**c**) A third tab shows the protein-protein correlations for the combined data or separately for the different study sets.

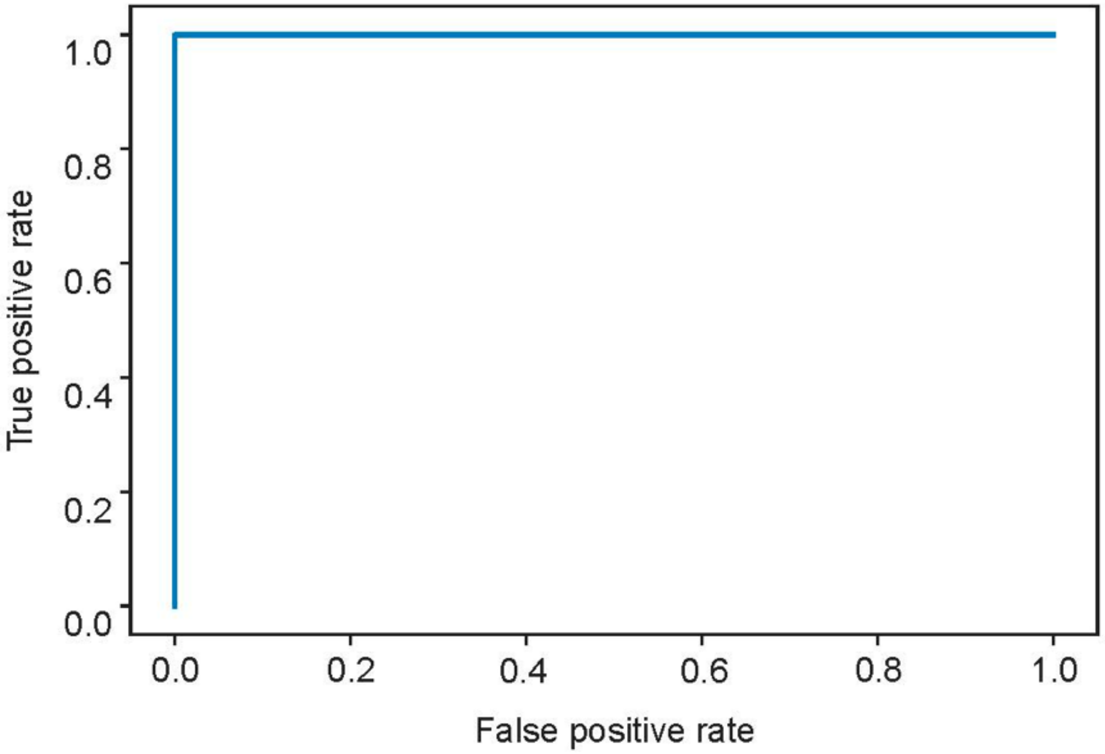

ESM Fig. 7 ROC curve for the training set. ROC curve illustrating the performance of the binary classification for infection status in the training group (Study 1). Sensitivity (true positive rate) is plotted against 1-specificity (false positive rate). The AUROC was 1.

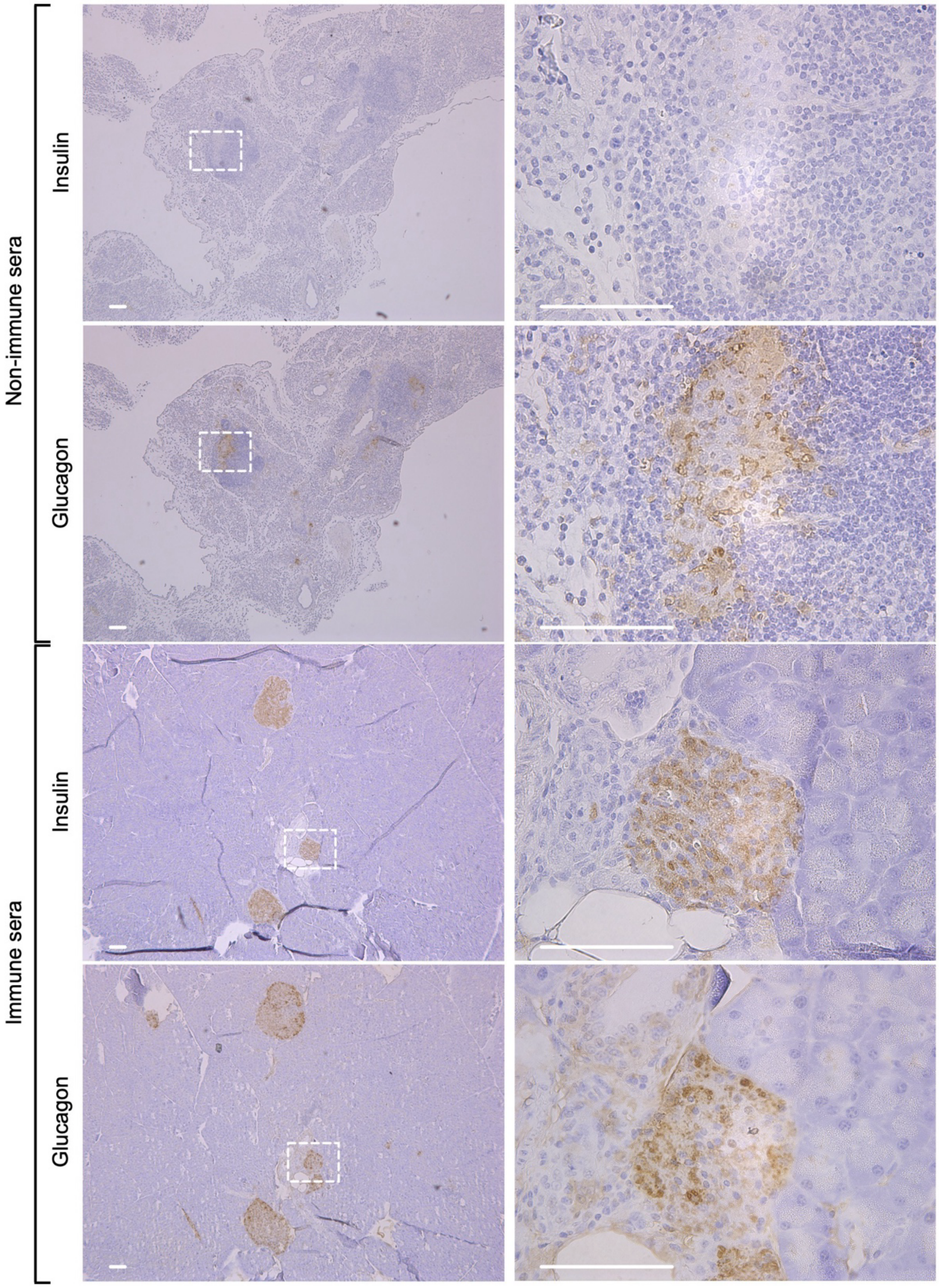

ESM Fig. 8 Pancreatic beta cell loss and exocrine pancreas damage in CVB3 infected SOCS-1-tg mice is prevented by an early intervention. SOCS-1-tg mice were infected with CVB3 (10^5 PFU CVB3, i.p., *n* = 14). On days 2 and 3 p.i., animals were treated with either non-immune (*n* = 8) or immune sera (*n* = 6). Animals were sacrificed on day 14 p.i. or at diabetes diagnosis. Representative images of insulin or glucagon antibody stained sequential pancreas sections from mice infected with CVB3 and treated with non-immune sera (upper panels) or immune sera (lower panels). Left column shows the wider landscape of the pancreas at 5x magnification, and right column shows the highlighted squares at 40x magnification. Positive areas are stained brown. Scale bar = 100 µm.

ESM References

[1] Flodstrom M, Maday A, Balakrishna D, Cleary MM, Yoshimura A, Sarvetnick N (2002) Target cell defense prevents the development of diabetes after viral infection. Nat Immunol 3(4): 373-382. 10.1038/ni771
[2] Serreze DV, Dwyer JR, Racine JJ (2024) Advancing Animal Models of Human Type 1 Diabetes. Cold Spring Harb Perspect Med 14(10). 10.1101/cshperspect.a041587
[3] Stone VM, Hankaniemi MM, Laitinen OH, et al. (2020) A hexavalent Coxsackievirus B vaccine is highly immunogenic and has a strong protective capacity in mice and nonhuman primates. Sci Adv 6(19): eaaz2433. 10.1126/sciadv.aaz2433
[4] Dodig-Crnkovic T, Hong MG, Thomas CE, et al. (2020) Facets of individual-specific health signatures determined from longitudinal plasma proteome profiling. EBioMedicine 57: 102854. 10.1016/j.ebiom.2020.102854
[5] Dahl L, Hagelin H, Hong M-G, Bendes A, Schwenk J (2024) Schwenk-Lab/ProtPQN: v1.0.1. In. Zenodo
[6] Rosenblatt F (1958) The perceptron: a probabilistic model for information storage and organization in the brain. Psychol Rev 65(6): 386-408. 10.1037/h0042519
[7] Paszke A, Gross S, Massa F, et al. (2019) PyTorch: An Imperative Style, High-Performance Deep Learning Library. arXiv

ESM Tables (separate file)

ESM Table 1.: Comparison of protein levels in CVB3 infected vs control mice at different time points (T-test and fold changes).

ESM Table 2.: *P*-values from GAM model used to assess linear and non-linear relationships between protein levels and the number of days p.i.

ESM Table 3.: Accuracy of mouse model to predict infection status on different days p.i.
